# Spatial and Temporal Field-Scale Accuracy Assessment of a Multi-Sensor Spade for In Situ Soil Diagnostics: Performance and Limitations of the Stenon FarmLab for Precision Agriculture

**DOI:** 10.3390/s25247430

**Published:** 2025-12-06

**Authors:** Görres J. Grenzdörffer, Jonas S. Wienken, Alexander Steiger

**Affiliations:** Department for Geodesy and Geoinformatics, Faculty for Agriculture, Civil and Environmental Engineering, University of Rostock, Justus-von-Liebig-Weg 6, 18059 Rostock, Germany; jonas.wienken@uni-rostock.de (J.S.W.); alexander.steiger@uni-rostock.de (A.S.)

**Keywords:** proximal soil sensing, sensor reliability, Stenon FarmLab, soil, nutrients, spatial variability, in situ soil analysis

## Abstract

**Highlights:**

**What are the main findings?**

- While the Stenon Famlab proximal soil sensing device reliably measured physical soil properties such as temperature and texture, chemical parameters like Nmin, phosphorus, and SOC showed poor temporal stability and low spatial correlation.
- Cluster and semivariogram analyses revealed stable spatial patterns for some parameters (e.g., pH), supporting limited potential for management zone delineation.

**What is the implication of the main finding?**

- The investigated 2022/23 version of the Stenon FarmLab is not yet suitable for precision nutrient management, but has potential with improved calibration and environmental compensation algorithms.

**Abstract:**

Real-time, in situ soil diagnostics are increasingly relevant for precision agriculture, but their efficacy under varying field and climatic conditions remains underexplored. This study assesses the 2022/23 version of the Stenon FarmLab, a multi-sensor soil analysis tool, over a 10-month period and across 1187 measurements on six fields (five cropped, one grassland) in northeast Germany. Despite the common approach of comparing a field sensor against lab results, in this paper, the FarmLab’s outputs are benchmarked using various approaches, such as time series, correlation, and geostatistical analysis, to fully evaluate the temporal and spatial stability and alignment with known soil heterogeneity. While physical soil parameters such as temperature and soil texture showed robust detection accuracy, key agronomic metrics—including mineral nitrogen (Nmin), soil organic carbon (SOC), and phosphorus—exhibited poor temporal consistency and low correlation with expected spatial patterns. Measurement errors and high sensitivity to weather conditions restrict data quality, particularly under frost and drought. Spatial clustering of more temporally stable parameters (e.g., pH, soil texture) allowed for limited zone delineation. We conclude that while the FarmLab shows partial potential for on-site soil sensing, significant limitations in nutrient measurement reliability currently prevent its use in operational precision agriculture. Enhancements in sensor calibration, environmental compensation, and software are needed for broader applicability.

## 1. Introduction

Current information on plant-available nutrients and soil pH in arable land is a prerequisite for determining nutrient requirements and thereby enabling effective fertilization management by farmers. The nutrient status of agricultural fields is traditionally assessed through soil sampling followed by chemical analysis in a laboratory. However, chemical laboratory analyses are time-intensive, costly, and require meticulous handling of samples—particularly nitrogen samples—which necessitates cooling during storage and transport [1,2,3]. To geolocate the samples and derive outputs such as variable rate treatment maps, the sampling locations are recorded in the field using GNSS.

Nitrogen (N) in the soil holds central importance in agriculture, being essential for plant growth and yield. However, significant N leaching can occur during the winter months due to precipitation and reduced plant activity. This leaching often results in N being displaced into deeper soil layers, rendering it inaccessible to plants during the growing season [4]. In accordance with seasonal changes of soil nutrients, several publications have shown that management zones are not static [5,6,7] and should change over time, and therefore, affordable multitemporal data of soil nutrients are required. Over recent years, various proximal soil sensing technologies have been developed to measure plant-available nutrients and pH levels more effectively [8]. These include spectral sensors, geoelectrical conductivity sensors, and electrochemical sensors, such as ion-sensitive field-effect transistors (ISFETs), which aim to provide higher precision measurements [9]. An alternative sensor-based approach to conventional soil sampling is the Stenon soil analysis spade, a portable device designed for direct, on-site measurement. This device has been certified by the German Agricultural Society (DLG) for its accuracy in measuring mineral nitrogen (Nmin) content and soil moisture [10]. Additionally, the device provides information on other nutrients, including phosphorus (P), potassium (K), magnesium (Mg), as well as pH levels and soil organic carbon (SOC). However, as these additional parameters are not certified by the DLG, they are marked as “beta” and are to be used “at the user’s own risk”.

Several studies conducted in recent years suggest that the measurement results from the Stenon device are often difficult to compare to conventional soil samples [11,12,13,14,15,16]. The observed discrepancies are likely attributable to several factors, including the high micro variability of soil and differences in measurement depth—conventional samples extend to depths of up to 30 cm, whereas the Stenon spade typically measures only the surface layer (up to 10 cm).

In summary, previous scientific comparisons indicate that the Stenon FarmLab does not sufficiently align with laboratory measurements of Nmin contents. Nonetheless, the device offers significant potential for capturing various soil properties that are not addressed through simple comparison with laboratory values. To explore the full potential of the Stenon device in the 2023 version or any other soil proximal sensor system and to assess its broader application potential, this study seeks to address the following research questions:

- Which parameters can be reliably and repeatedly measured with the Stenon device, and which cannot?
- Are the measurements and temporal trends of individual parameters provided by the Stenon device realistic?
- What role do weather conditions (e.g., temperature, dryness, frost) play in the measurability and interpretability of the derived parameters?
- To what extent do management practices and varying soil conditions affect the measurements?
- Is it feasible to capture spatial soil patterns using the Stenon device, and if so, at what distances should measurements be conducted?

To address these general questions about the quality assessment of proximal soil sensor systems, various measurement strategies across different locations and extended timeframes are necessary:

1. Measurements at a single point over a defined period of time to investigate temporal variability and reproducibility.
2. Measurements at multiple points within a field over a given time frame to assess spatial heterogeneity and temporal trends of various parameters.
3. Measurements across different fields to evaluate the impact of site-specific conditions, management strategies, and fertilization practices.
4. Measurements at different locations within a field over a defined period to (geo)statistically analyze the influence of measurement spacing on spatial patterns.

## 2. Materials and Methods

### 2.1. Site Descriptions

**Figure 1:**
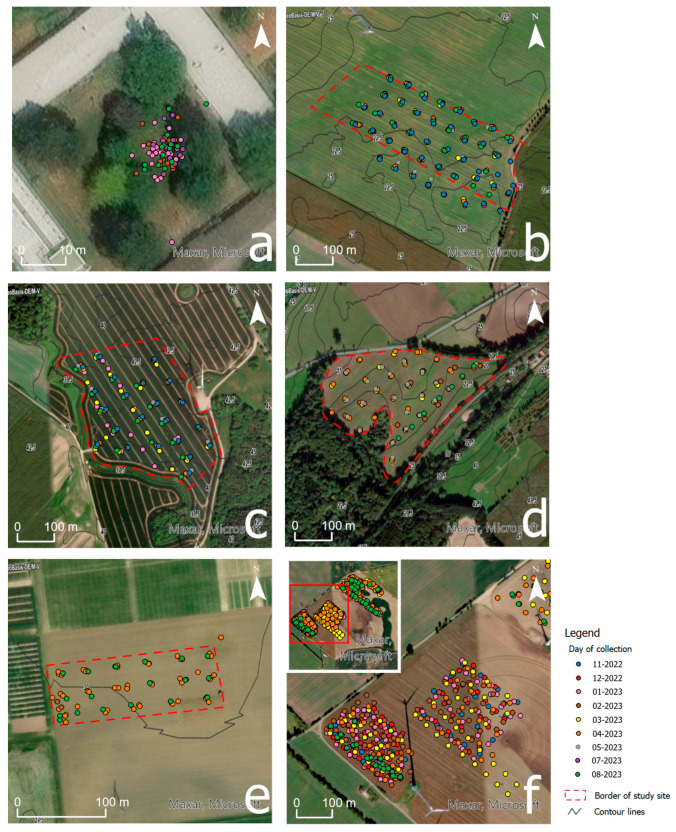
Aerial photographs of the study areas with contour lines drawn in, as well as measurements taken as dots colored by month in the surveyed areas. The red dashed line shows the boundaries of the study areas: (**a**) AUF; (**b**) Groß Schwiesow; (**c**) Dummerstorf; (**d**) Teterow; (**e**) Groß Lüsewitz; (**f**) Kassow.

The investigation of the topsoil with the Stenon device was conducted on five agriculturally managed fields and one grassland area. All six locations are situated within the district or city of Rostock, Mecklenburg-Vorpommern, Germany. Aerial images of each field, along with corresponding contour lines, are presented in Figure 1. The specific study areas within each field are outlined with red dashed lines.

Location (a) represents a special case compared to the other fields. It is located within the campus of the Faculty for Agriculture, Civil and Environmental Engineering (AUF) of Rostock University. Unlike the other fields, it is not used for agricultural purposes but instead is a grassland area situated within the courtyard of the AUF. Care was taken to select a site where no interventions affecting soil quality had been undertaken.

Detailed information about the agriculturally managed fields is provided in Table 1. The fields were chosen based on their specific site conditions and management practices. As a result, varying outcomes are expected during the investigations, which should nonetheless be representative of the respective field’s characteristics.

Fields (b) to (f) were fertilized according to crop type and management strategy. An overview of the fertilization measures and their respective application times for each field is provided in Table 2.

### 2.2. The Measurement Device—Stenon FarmLab

The investigation of topsoil on the fields was conducted using the FarmLab device from Stenon GmbH (Potsdam, Germany). FarmLab is a portable multi-sensor system that can only be rented alongside a cloud-based solution. The device resembles a spade, with its lower end equipped with various optical and electrical sensors to measure different soil parameters. The device is operated via a touch control panel located on the handle. Additionally, it features a GNSS module for geolocating measurements and a WiFi module for connecting the FarmLab to the internet, enabling real-time access to measurement data in the cloud solution.

Before starting a measurement campaign, the device requires a warm-up phase of several minutes. After this initialization, the optical sensor must be calibrated using the provided calibration cap. Once calibration is complete, the spade-like device is inserted into the topsoil at the designated sampling location, and the measurement process is initiated via the touch control panel. The sensors employ two main measurement principles: electrical impedance spectroscopy and optical VIS/NIR spectrometry [18]. The electrical impedance sensor, located at the tip of the sensor system, collects data across low, radio, and microwave frequency ranges for a measurement volume of approximately 19 cm^3^. The optical VIS-NIR spectrometer is located on the opposite side of the measurement head and operates with an illuminated measurement area of approximately 0.9 cm^2^. Additionally, the soil temperature is recorded.

Each FarmLab measurement comprises three individual readings taken within an area of about one square meter. Averaging these readings helps to reduce micro variability and ensures a representative value for the respective site. After each sampling or measurement, the sensors must be cleaned. The FarmLab device can upload measurement data in real-time to the Stenon Cloud via WiFi. If no internet connection is available, the device can store up to 1000 measurements locally, which can be synchronized later. Based on an algorithm developed by Stenon, the three sub-measurements are combined to produce a final result and derive a wide range of soil parameters. The results are online accessible via the Stenon cloud solution. In S1. Online Resource 1, the parameters and measurement ranges that can be captured by the FarmLab device are displayed. In addition to the parameters shown in S1. Online Resource 1, the system also provides the soil texture class, a timestamp, and the coordinates of the measurement location in the WGS 84 coordinate system. The device used in this study was identified as Stenon Device #297 (p3).

### 2.3. On-Site Data Collection

The observation period for the study ranged from 9 November 2022 to 31 August 2023. The investigation period was originally designed to capture the nitrogen dynamics in the soil during winter and spring, leading up to the harvest. The plan was to sample the topsoil of the agricultural fields once per month. Depending on the size of the study area (6–23 ha), vegetation density, and vegetation height, the measurements required between three and five hours per field. Table 3 provides a summary of the investigated fields and the corresponding measurements conducted each month. Fields (b), (c), and (f) were studied throughout the entire observation period, while measurements on fields (a), (d), and (e) were only carried out starting in 2023. During the winter months (December to February), gaps in measurements occurred for fields (b) and (c) due to soil frost, which made it impossible to sample the topsoil during the designated periods. Furthermore, gaps were observed across all agricultural fields ((b) to (f)) during the summer months (May to July). These gaps resulted from excessively dry soil conditions in the summer of 2023, which rendered topsoil sampling using the FarmLab device difficult to impossible in many parts of the fields.

Over a period of four months (May–August 2023), measurements were conducted on location (a) to assess the variance and repeatability of the measurement method. For this purpose, daily or bi-daily measurements were taken at a selected location within the courtyard of the AUF, which was not fertilized or treated with additional nutrients (see Figure 1a).

Two different approaches were used to sample the agricultural fields. One approach involved measuring the topsoil parameters of the fields in a uniform grid. This approach was applied to fields (b) to (e). For its implementation, square tiles served as references, with their centers subsequently sampled. On fields (b), (c), and (d), the underlying tiles had side lengths of approximately 72 m, corresponding to double the tramline spacing. The tiles for field (e) were chosen with a side length of about 40 m, which was denser due to the smaller field size. This created a measurement pattern that remained largely consistent across multiple campaigns, enabling conclusions about the temporal behavior of parameters for this specific area over a desired period (see Figure 1b–e). In contrast, the second approach involved irregularly collected measurement points (see Figure 1f). For this purpose, for each survey, other locations were selected within the study area. The selection of points was determined individually for each measurement based on the conditions prevailing on the day of sampling. This approach has the advantage of creating a very dense “point cloud” after several campaigns, preferable for geostatistics, and allows conclusions to be drawn about the spatial heterogeneity of the site, see Table 4 for a summary of the different sampling approaches.

Figure 1 shows the data collection strategies employed in the respective study areas. The aerial images are supplemented with contour lines, and the areas are outlined in red. The measurements conducted are displayed as points, with the color of the points corresponding to the month in which they were collected. Figure 1a illustrates the measurements for evaluating the precision of FarmLab on the grounds of the AUF. The measurements in field (a) were always carried out around the same point. However, due to the limited accuracy of the GNSS receiver, the observations appear to be taken at different locations. Figure 1b–e show fields with measurements carried out with a regular sampling pattern. Figure 1f shows the two southeastern subfields of the study area (f) where the randomized sampling pattern was applied.

## 3. Results

### 3.1. Time Series Analysis for Location (a)

On location (a), a total of 74 measurements were conducted between 3 May and 23 August 2023. In rare cases when the soil was too dry to obtain a measurement, additional water was supplied. The objective of this measurement campaign was to assess the accuracy, repeatability, and variability of selected soil parameters over an extended time period at a single measurement point. Since the location did not receive fertilizers, neither in organic nor in mineral form, abrupt changes in the measured soil nutrients were not expected. The statistically and agronomically evaluated parameters include plant nutrients (Nmin, P, K, and Mg), as well as soil properties such as SOC, soil moisture, pH, and soil texture (Soil Texture Class).

While seasonal fluctuations were anticipated for soil nutrients and pH, soil texture and, to some extent, SOC content were expected to remain relatively stable during the time period unless extreme weather events occurred [19,20]. However, long-term changes in agricultural management practices may change the SOC significantly [21]. In contrast, soil moisture content and soil temperature were expected to be more influenced by external factors such as precipitation and ambient temperature, both of which were considered in this investigation [22]. Table 5 summarizes the descriptive statistics for the parameters measured on location (a), including Nmin, P, K, Mg, SOC, moisture content, pH, and soil temperature.

**Figure 2:**
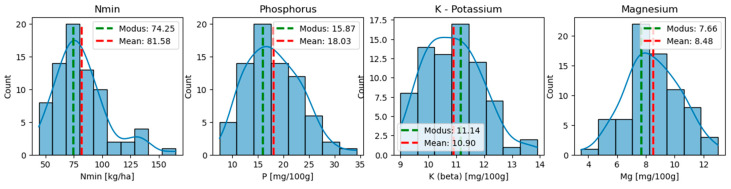
Histograms of location (a) for Nmin, phosphorus, potassium, and magnesium measurements with modus (green) and mean (red).

The nutrient parameters exhibited varying degrees of variability. K, for instance, showed moderate standard deviation (σ = 1.02 mg/100 g), while nutrients such as Nmin (σ = 23.44 kg/ha), P (σ = 5.26 mg/100 g), and Mg (σ = 1.89 mg/100 g) displayed high variability. The distribution analysis of these values (see Figure 2) revealed that the modal values of all nutrients were below their means, indicating a positive skewness in their distributions. This skewness can be attributed to natural processes in the soil that result in this uneven distribution [23]. Nmin showed particularly high deviations due to outliers, with values reaching up to 165 kg/ha—74.25 kg/ha above the modal value. In S2. Online Resource 2, results of Shapiro–Wilk tests on parameters Nmin, phosphorus, potassium, and magnesium of location (a) are also provided.

**Figure 3:**
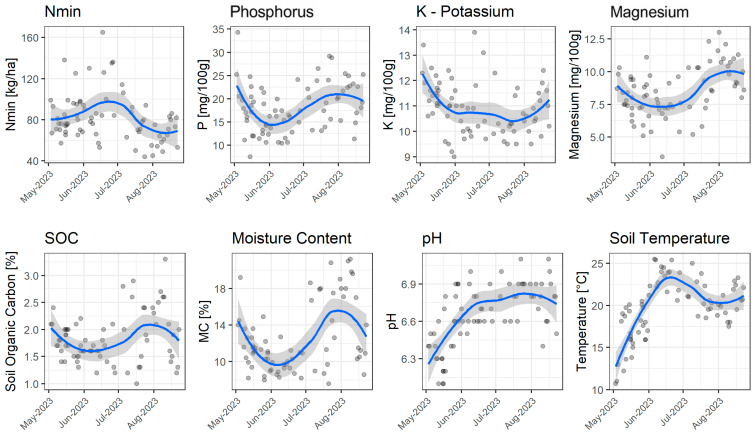
Scatterplots of location (a) with Nmin, phosphorus, potassium, magnesium, SOC, moisture content, pH value, and soil temperature with trendline and standard deviation.

SOC exhibited surprisingly high standard deviations (σ = 0.46), reflecting significant variability that increased over the summer, Figure 3. The temporal development of SOC, as well as its increased summer variability, appeared to correlate with soil moisture levels. The pH value (σ = 0.25) showed minimal fluctuation. It increased between May and June and remained stable throughout the summer. This trend, at least superficially, appeared to align with the progression of soil temperature, which is unusual, as literature suggests that pH tends to decrease during the summer months [24].

The determined soil texture by FarmLab revealed high consistency with the predominant texture of the topsoil in the courtyard. In 72 out of 74 measurements, a “sandy soil” was recorded, which aligns with the measured soil texture. When broken down into the soil groups (BG) of the VDLUFA classification system, these 72 measurements were distributed as follows: sand (BG 1) in 16 measurements, slightly loamy sand (BG 2) in 46 measurements, and strongly loamy sand (BG 3) in 10 measurements [25]. In two measurements, the texture was classified as “loamy” (BG 2 and BG 3), likely due to increased soil moisture at the time of measurement. The “loamy” soil texture and the highest soil moisture levels (20.8% and 21%) were recorded on 23 July and 6 August 2023, shortly after days of significant rainfall.

**Figure 4:**
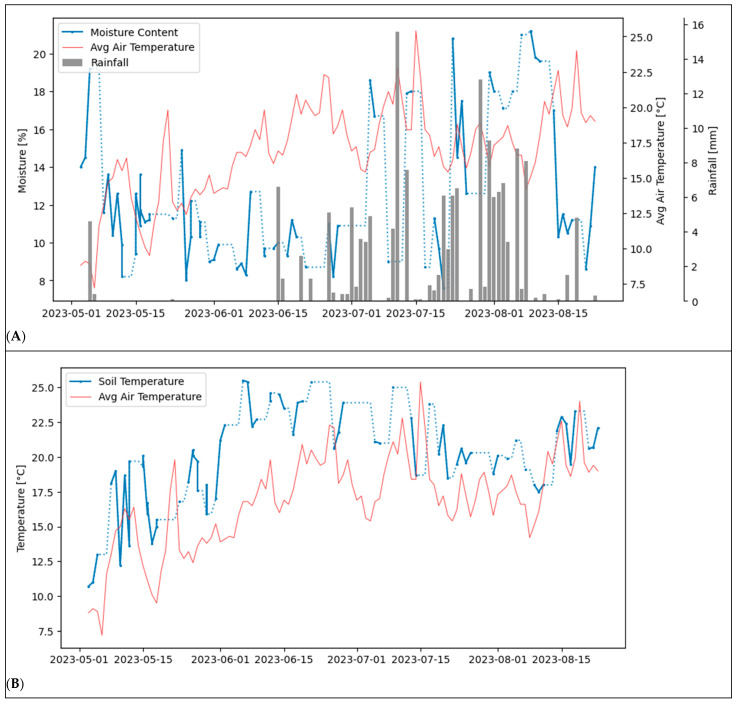
Influence of climatic conditions on the measurements on location (a):(**A**) comparison of soil moisture content (Stenon FarmLab), average temperature, and daily rainfall (source for avg. temperature [26]; source for rainfall (C. Frank, Personal Correspondence, 9 August 2024)); (**B**) measured soil temperature (Stenon FarmLab) and average air temperature (source for avg. air temperature [26]).

Moisture content and soil temperature were strongly influenced by climatic conditions. Figure 4A illustrates the relationships between moisture content, average air temperature, and daily precipitation levels, compared to data from a local weather station. The missing values due to days without measurements taken are displayed using dotted lines. For these values, the last measured value is kept to bridge the potential gaps between two measurements. As expected, soil moisture exhibited fluctuations driven by precipitation events and high summer temperatures, particularly from late June to early August. Soil temperature and ambient air temperature data from the weather station follow the same path, as expected, and show seasonal fluctuations depicted in Figure 4b. The fluctuations in soil moisture values during the rain-free episode between May and mid-June can be explained by local irrigation measures to ensure the functionality of the Stenon FarmLab. The topsoil temperatures averaged 19.74 °C, with a standard deviation of 3.45 °C.

### 3.2. Correlation Analysis in Relation to Different Site Characteristics and Management Practices (Fields (b)–(f))

**Figure 5:**
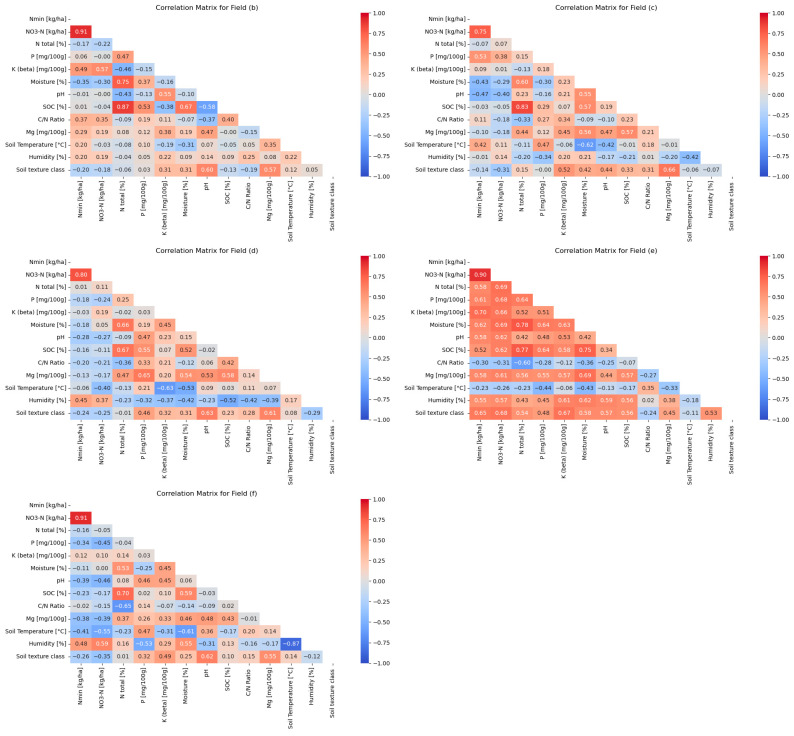
Correlation matrices of selected soil parameters measured with the Stenon FarmLab at the five agricultural fields (b)–(f).

The correlation analysis of nutrient and soil parameters across the agricultural fields (b)–(f) reveals significant variability in the correlations between measured variables. Each field exhibits a distinct correlation pattern shaped by site-specific factors such as soil type and management practices, including crop rotation and fertilization intensity. These patterns reflect the specific interactions among the parameters influenced by local conditions, see Figure 5.

Previous studies (e.g., ref. [27]) have long demonstrated that nutrients like N, P, K, and Mg show considerable variation in correlation strength and direction across fields. This is not surprising given the differing temporal dynamics of soil nutrients in terms of plant uptake, leaching, and soil fixation.

A closer examination of fertilization intensity and its impact on correlations reveals notable differences. Field (e), which is conventionally managed and planted with rye, exhibits a strong correlation between N values and other nutrients, suggesting the influence of intensive fertilization shortly before sampling with the Stenon FarmLab. This interpretation is supported by the relatively high quantities of N fertilizers applied, including urea and NPK fertilizer. In contrast, field (d), managed organically, displays moderate correlation patterns, likely due to the more restrictive fertilization strategies typical for organic farming.

N, P, K, and Mg levels, as well as pH values, undergo substantial and abrupt changes following mineral or organic fertilization. Apart from this, from an agronomic perspective, certain seasonal trends in nutrient levels are expected. For instance, soil Nmin content tends to peak in the autumn and gradually decrease through winter due to leaching, then increase again in the spring with fertilization and the onset of mineralization. As plant growth accelerates, the availability of soil N diminishes until harvest. Similarly, seasonal fluctuations in P, K, and Mg levels are expected in the absence of additional fertilization. For P, temperature-dependent solubility changes can influence plant-available P [28,29]. pH values can also exhibit slight seasonal variations. Factors influencing pH include the amount and timing of fertilizer application, organic matter content, root and microbial activity, and soil moisture. Typically, pH values are lower in summer and early autumn and increase with rising soil moisture [24]. SOC levels are generally stable over time, with changes occurring primarily in the long term [20]. However, SOC measurements can exhibit short-term or seasonal fluctuations, often due to micro-variability [30].

**Figure 6:**
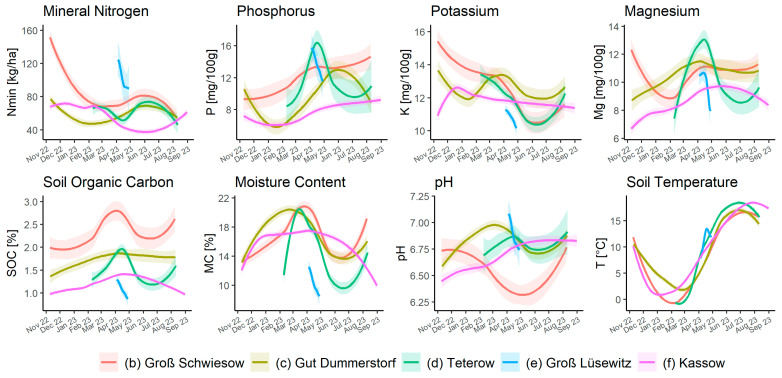
Site comparison of soil parameters over time for fields (b–f) was created using R’s LOESS smoothing method [31], which fits local regressions weighted by the distance of neighboring points. The shaded area represents the 95% confidence interval around the fitted curve.

The time series analysis of soil parameters (see Figure 6) illustrates the site-specific and seasonal variability of the measured values for fields (b), (c), (d), and (f). The temporal aspects complement the results of correlation matrices and demonstrate that parameters like Nmin and soil moisture are highly responsive to seasonal and weather fluctuations, which in turn influence their correlations with other nutrients. The temporal stability of pH values explains their weaker correlation with other factors, making them a reliable basis for long-term soil analyses. The dependence of soil moisture and soil temperature on seasonal weather influences is evident.

### 3.3. Derivation of Management Zones—Cluster Analysis for Field (b)

**Figure 7:**
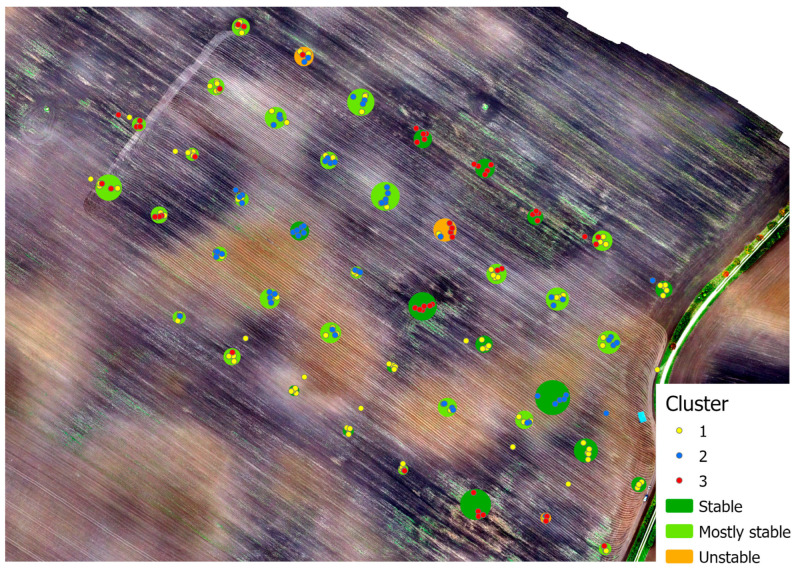
Multivariate cluster analysis (3 clusters) based on temporally “constant” soil parameters (SOC, pH, relative soil moisture, soil texture) over six campaigns on field (b), background: RGB-orthomosaic acquired 28.9.2022. Spots are classified into three categories by the number of clusters per spot (stable = 1, mostly stable = 2, and unstable = 3).

A multivariate K Means algorithm cluster analysis was conducted on field (b). Over a period of approximately ten months, during which 239 samples were collected at approximately the same spots in six campaigns, three stable clusters were identified (yellow, red, and blue markers in Figure 7). These clusters reflect the variability of the soil and are defined by stable parameters such as SOC, pH, relative soil moisture, and soil texture [20]. The temporal stability of these clusters, determined by the number of clusters within a spot, indicates that these parameters exhibit high consistency at most sampling points. This consistency can be attributed to the fundamental physical and chemical properties of the soil, providing a basis for the derivation of management zones. The clustering relies on key soil-specific characteristics, particularly soil texture, humus content, and topography. These parameters are critical because they influence water and nutrient availability and determine the spatial distribution of soil fertility within the field. Studies confirm that the spatial variability of soil properties enables targeted management in the form of management zones, thereby improving the efficiency of agricultural practices [32,33,34,35].

The cluster analysis is visually represented by the markers in Figure 7. The different colors of the markers represent individual clusters, which differ based on the stable soil parameters (SOC, pH, relative soil moisture, and soil texture). These color-coded points on the image illustrate the spatial distribution of management zones within Field (b). The color differences highlight that certain areas of the field share similar properties, forming the basis for dividing the field into management zones. The spatial distribution of the clusters, depicted as colored markers, further illustrates the heterogeneity of the soil. Markers belonging to specific clusters are often grouped together, indicating more homogeneous soil conditions in these regions. The delineation of individual clusters also corresponds well to the top soil color, as shown in the aerial image, acquired after harvest and soil cultivation on 28 September 2022. For instance, many blue markers are found in light brown areas of the aerial image, corresponding to elevated areas with mineral soils (see Figure 1b), while many red markers are located in local sinks (darker, wetter areas). Yellow markers are frequently observed in the transitional zones between the other two clusters. Markers that are more dispersed or located between different cluster colors suggest transitional zones where soil parameters are expected to be less stable. This visualization of cluster distribution not only underscores the spatial heterogeneity of the soil within the field but also highlights its potential for site-specific farming practices.

### 3.4. Geostatistics for Field (f)

**Figure 8:**
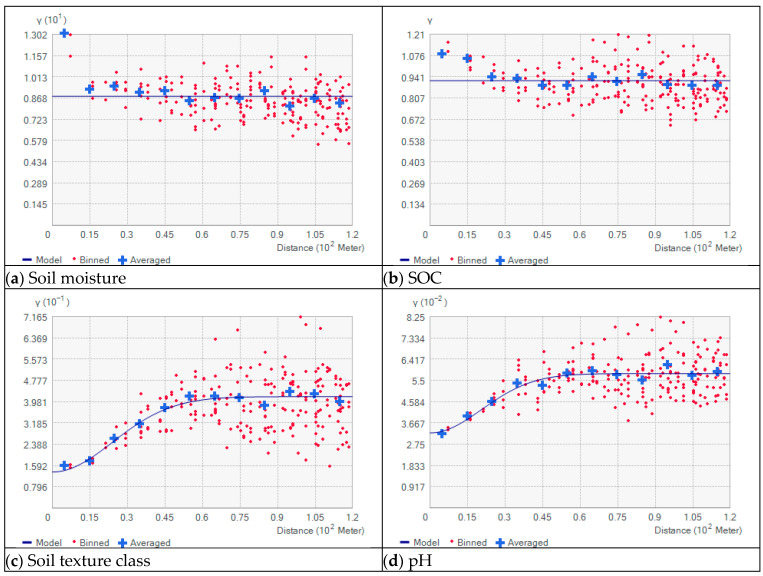
Semivariograms for different soil parameters on field (f) based on 537 observations: (**a**) soil moisture, (**b**) SOC, (**c**) soil texture class, and (**d**) pH-values.

Geostatistical methods provide a detailed examination of the spatial variability of soil properties by analyzing the correlation between points as a function of their spatial distance. In this context, semi-variograms offer valuable insights into the spatial continuity and degree of heterogeneity for field (f). The analysis of semi-variograms aims to identify patterns and trends in soil properties, focusing on parameters such as soil moisture, pH, soil texture, and SOC [36,37]. Different soil parameters exhibit varying spatial variability. This is also referred to as range or lag distance in variogram analysis. At the same time, the sampling distance—unfortunately—has a decisive influence on the statistically derived variability of individual soil parameters [38]. For this reason, a very high spatial resolution of soil sampling is required to reliably estimate the spatial variability of different soil parameters. Soil nutrient contents change over time and therefore cannot be analyzed geostatistically in a meaningful way with the multitemporal data set for field (f). The following figure shows the semi-variograms of SOC, soil moisture, soil texture, and pH for field (f). The mean point spacing between the 537 points obtained over an area of approximately 23.6 ha in eight measurement campaigns is 21 m. A lag was uniformly specified with 10 m and 120 m as the maximum lag distance, see Figure 8.

**Figure 9:**
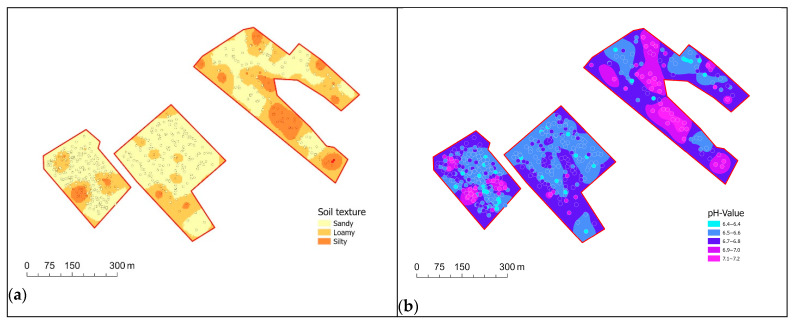
Interpolated maps for data collected on field (f) of (**a**) soil texture of field (f) (simple kriging), based on 537 measurements, displayed as overlay RMSE = 0.42, and (**b**) soil pH (simple kriging), based on 537 measurements, displayed as overlay RMSE = 0.19.

As expected, the semi-variogram of soil moisture shows no dependence of the variance on the distance between the measurements. This is due to the fact that soil moisture is not only influenced by soil properties, but is also dependent on weather conditions. Since, as already determined in the time series analysis for location (a), the measurement of SOC is strongly influenced by soil moisture and was not decoupled. Accordingly, the semi-variogram of SOC also shows no spatial correlation. The situation is different for the parameters pH and soil texture. Both soil parameters show significant trends, i.e., the (semi)variance increases with greater distance. The average ranges of the variograms, using a Gaussian model and allowing for an automatically computed nugget effect for the two soil parameters, are comparable. For the soil texture, the range, i.e., the point at which the sill is reached, is 60 m, whereas for the pH value, it is 72 m. Figure 9a,b show the derived maps using simple kriging and based on the empirical semivariograms presented above of the soil texture class and the pH value.

### 3.5. Error Messages and Their Impact on Measurement Efficiency

**Figure 10:**
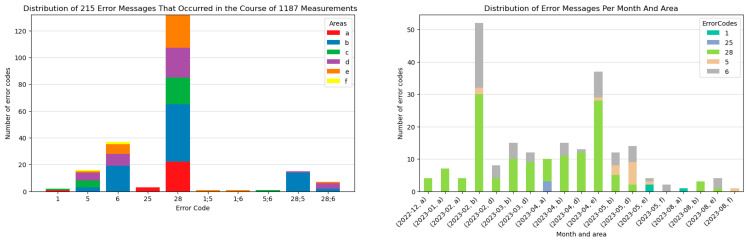
Distribution of 215 error messages that occurred in the course of 1187 measurements (**left**) and distribution of error messages per month and area (**right**): 1: air measurement with calibrated device, 5: soil measurement with air gap between EIS and soil, 6: soil measurement with air gap between optical sensors and soil, 25: noise in EIS spectra, 28: vegetation [39].

During the measurement campaigns, error messages occurred frequently. Among the 27 campaigns conducted across agricultural fields (b–f), only five campaigns were completed without any errors ((b): 2022-11; (d): 2023-08), (f): 2022-11, 2023-03, 2023-07). Notably, in field (d) during the August 2023 campaign, only seven measurements could be conducted due to the unharvested rye. Across all fields and campaigns, Figure 10 (left) illustrates that Error Code 28 was the most common, occurring 132 times during the 1187 measurements. Error Code 28 is triggered by vegetation residues detected by the sensors. Potential causes for this error, as described by [39], include fresh or partially degraded leaves or crops, straw, etc., in front of sensors; vegetation in front of sensors; and organic fertilization and a thin moss layer, especially on top of sandy soils.

When this error occurred, the sampled hole was inspected for vegetation residues. If such residues were identified, they were removed, and the sampling was repeated. However, it was noted that not all instances of Error Code 28 could be attributed to the listed causes. The second most frequent error was Error Code 6, which occurred 37 times. This error is associated with air gaps between the optical sensors and the soil during measurements. Potential causes for Error Code 6, as outlined by [39], include the tap between soil and optical sensors, the sensor head not fully submerged, or very dark/very bright soils.

A notable spike in error messages occurred during the February 2023 campaign on Field (b) (Figure 10, right). An analysis of the measurements from this period revealed a pattern linked to environmental conditions. The average air temperature during these measurements was 3.73 °C, while the average soil temperature was −0.12 °C. Consequently, the topsoil was frozen in many areas, significantly hindering the insertion of the Stenon FarmLab device. Additionally, the frozen soil contributed to an increased occurrence of Error Code 6, as the soil did not adequately surround the sensors. Similarly, vegetation on the frozen surface exacerbated the occurrence of Error Code 28, as it was challenging to remove under these conditions.

To mitigate potential error messages, it is crucial to prepare the three sampling sites adequately before measurements. During the campaigns, a promising approach to address Error Code 28 involved lightly scraping the top layer of soil with a rake to remove plant residues, grass, or moss. This preparation is highly recommended as the frequency and type of errors significantly impact the duration of a measurement campaign. For example, an analysis of the time intervals between consecutive measurements on Field f reveals that a smooth measurement typically takes 03:37 min (see Table 6). On average, 60 measurements were conducted per campaign in field (f) (see Table 3), which under ideal conditions would require a total duration of 03:37:00 h. However, based on our experience, approximately one in every four measurements encounters at least one error. Depending on the type of error, corrective actions must be taken. These include cleaning the sensors, locating a new sampling spot, and preparing it with a rake. For a basic error requiring a single retry, the average measurement duration increases to 05:26 min. In the case of 12 faulty measurements (one-fourth of the 60 measurements), this results in a total duration of 04:04:08 h, an additional 27:08 min compared to error-free conditions. More complex errors, such as requiring recalibration of the sensors, significantly extend this time frame further.

## 4. Discussion

### 4.1. Time Series Analysis (Location (a))

The results of the time series analysis on location (a) demonstrate significant variability of the measured nutrients over time, reflecting a broad range of values, which cannot be fully explained by agronomical expectations. Soil parameters, such as SOC and pH, which should be stable over time, also display significant seasonal changes, which are not in accordance with findings in the literature, e.g. [19,20], which indicated that SOC and pH exhibit minimal seasonal fluctuations under stable climatic conditions.

The observed correlation between SOC and moisture content, with a coefficient of r = 0.85, highlights the remaining and uncorrected influence of soil moisture on the measurement of SOC, particularly under high moisture conditions during the summer months. This strong relationship is consistent with findings of [40], who also reported a robust correlation between organic matter and soil moisture.

The Nmin values exhibit some extreme outliers. These exceptionally high Nmin readings do not correspond to typical seasonal fluctuations. Such variations may be triggered by external factors, such as heavy rainfall or N mobilization in the soil, leading to unexpectedly high values. Alternatively, these anomalies might suggest measurement inaccuracies by the FarmLab sensor, particularly given the uncharacteristic nature of such Nmin spikes on untreated fields. This indicates that the sensor may exhibit heightened sensitivity to N fluctuations driven by temporary environmental conditions.

The fluctuations in moisture content and soil temperature clearly reflect their dependence on climatic factors such as rainfall and ambient temperature. These findings align with prior knowledge that high summer temperatures combined with a lack of rainfall can lead to rapid drying of the topsoil. The measurements suggest that FarmLab accurately records these dynamic parameters. Similarly, the fluctuations in soil temperature, which strongly correlate with seasonal ambient temperatures, match expectations. Soil temperature influences processes such as microbial activity and mineralization rates, which in turn affect nutrient availability [41]. This correlation further supports the accuracy of FarmLab measurements regarding soil temperature.

Given the likelihood of measurement errors in this case study, particularly concerning Nmin, additional validation measures are necessary to minimize such inaccuracies. Implementing stricter control protocols and cross-referencing Nmin measurements with standard laboratory methods is recommended to ensure reliability, especially in the presence of outliers [15].

### 4.2. Correlation Analysis (Fields (b)–(f))

The results of the correlation analysis confirm that management practices and fertilization strategies have a significant impact on the availability and interaction of nutrients in the soil. These findings are largely consistent with insights from the literature [42,43]. For instance, the conventionally managed field (e), which was intensively fertilized with N, exhibited a high correlation between N and other nutrients. This strong correlation aligns with earlier studies showing that conventional fertilization methods enhance nutrient availability and lead to stronger nutrient binding [44]. The confirmation of such correlations by FarmLab measurements in field (e) supports the reliability of the sensor in depicting interconnected nutrient dynamics under conventional fertilization.

In contrast, measurements on the organically managed field (d) displayed only moderate correlations between nutrients. These weaker correlations are also consistent with expectations from the literature, which highlights that organic fertilizers in ecological systems promote a more uniform but slower release of nutrients, thereby reducing the availability and interdependence of nutrients [45]. The FarmLab results, therefore, align well with the anticipated effects of different management practices on nutrient dynamics.

The differences in soil types on fields (b) and (c) are also reflected in the correlations, matching findings from the literature. Loamy soils, such as those on field (c), demonstrate higher water and nutrient retention capacity compared to sandy soils, such as those on field (b). This difference is evident in the more stable correlations between moisture and nutrients in loamy soils [46,47]. This agreement with literature indicates that the FarmLab sensors can effectively capture interactions in different soil types and reflect the dependency of nutrient dynamics on soil texture.

### 4.3. Cluster Analysis (Field (b))

The cluster analysis on Field (b) identified three spatially and temporally stable clusters based on soil parameters such as SOC, pH, moisture content, and soil texture. These clusters, visually represented by color-coded markers, align with expectations regarding the variability of site-specific soil characteristics such as moisture and humus content (see Figure 7). The distinct delineation of zones demonstrates that soil-specific properties like texture and moisture were reliably captured for this field. These properties provide a solid foundation for the delineation of site-specific management zones [48]. The identification of stable soil-related clusters indicates that the FarmLab sensors can provide reliable information about the spatial heterogeneity of soil properties, consistent with established soil physics principles.

### 4.4. Geostatistics (Field (f))

The geostatistical analysis showed that the FarmLab recognized the spatial pattern of the soil texture and the pH value in the field (f) quite reliably. The data were collected over a longer period of time. This in turn suggests that the measurement of soil texture and pH is not or only minimally influenced by external weather conditions during the measurement. Due to the high point density, the range of soil heterogeneity could be determined. The observed ranges of 60 and 72 m are in agreement with values from the literature [38]. Additionally, [16] revealed that the values are also in agreement with the online multi-sensor from UGhent [49]. The SOC values are strongly influenced by the weather and, at the same time, show a high micro variance [50]. Consequently, it was not possible to derive a map of the SOC. New geostatistical methods, e.g., [51], may be helpful in the future.

### 4.5. Error Messages

A recurring issue with the FarmLab device was the frequent occurrence of error messages, particularly under extreme weather conditions such as frost or drought. These conditions posed significant challenges during measurement campaigns. Frozen soil made it impossible to insert the sensor without risking damage to the device, while dried-out topsoil in summer often prevented the sensors from penetrating the soil deeply enough or resulted in insufficient contact pressure between the soil and the sensors. This led to error codes such as “Air measurement with calibrated device” (Error Code 1), “Soil measurement with air gap between EIS and soil” (Error Code 5), and “Soil measurement with air gap between optical sensors and soil” (Error Code 6).

These error messages indicate that the FarmLab measurement device struggles under extreme conditions, revealing its operational limits. This suggests the need for additional modifications or alternative measurement techniques to enable reliable data collection throughout the year, even in challenging conditions. Reducing error frequencies would not only improve efficiency but also enhance the overall reliability of the data collected under diverse environmental scenarios.

## 5. Conclusions

The analyses carried out with the Stenon FarmLab on various agricultural areas provide a comprehensive picture of the potential of the Stenon FarmLab that goes far beyond a simple comparison with laboratory analyses. The results show that the soil nutrient values measured with the FarmLab are generally within the expected range; however, there were significant fluctuations between nearby measurements that clearly exceeded the expected micro variance. This makes the use of the 2022/23 version of the Stenon FarmLab unattractive for precision farming applications.

Despite these limitations, soil texture was almost every time classified correctly, and some temporally plausible seasonal trends were observed for certain soil nutrients and soil moisture. However, the measurements showed significant temporal fluctuations in SOC and some nutrients, although almost stable measured values were expected. The fluctuations in SOC are almost synchronous with the course of soil moisture. It is well known from the literature [40,52] that the optical VisNIR measurement of SOC correlates strongly with soil moisture. This means that the device may not sufficiently compensate for these influences, or that fluctuations in soil moisture affect the measured SOC values. This underlines the need for further investigation and improvement of the device functionality.

The frequent error messages on the control panel during the measurement campaigns prove that the measuring spade has a kind of warning system for gross measurement errors. In some cases, however, these messages were not comprehensible and therefore often resulted in manual examinations of the sampling points or repeated measurements, which can drastically reduce the performance per unit area.

One of the main weaknesses of the Stenon FarmLab when used in practice in Central Europe is its sensitivity to certain weather conditions. During periods of frost or extreme drought, measurements could not be taken as the device had difficulty penetrating frozen soils or gave error messages due to dryness. This leads to gaps in data collection during frost and drought periods, making it difficult to capture the full temporal development of soil parameters over longer measurement periods.

The device proved useful in capturing the upper soil layers, but this limited measurement depth may not be sufficient to provide a comprehensive understanding of deep soil processes relevant to agricultural use and fertilization. While some spatial variability in soil parameters such as SOC and soil texture was recorded, the overall accuracy of this data remains questionable due to the inconsistencies observed.

Based on the measurements (November 2022–August 2023) and results of this study, the 2023 version of the Stenon FarmLab cannot be recommended for widespread agricultural use for soils in northeast Germany. While the device is user-friendly and relatively inexpensive, the large spatial and temporal scatter in the measurements means that the results may not be reliable enough to make informed decisions about fertilization, soil quality, or water management. Further research and development are needed to optimize the device for practical use in agriculture and to ensure that it provides accurate and consistent results under a wider range of environmental conditions.

In summary, although the Stenon FarmLab (2023 version) offers potential as a soil analysis tool, significant improvements are needed in terms of measurement accuracy, sensitivity to weather conditions, and spatial accuracy before it can be confidently recommended for everyday agricultural use.

It is hoped that the current version of the Stenon FarmLab (2025), which features an additional UV sensor and significantly improved software, will have addressed these issues.

## Figures and Tables

**Table 1:** Site information on the study areas (soil type: [17]).

Field	Total Area [ha]	Predominant Soil Type	Cultivated Crop	Management Method
**a**	-	Loamy sand	-	-
**b**	10	Slightly loamy sand to clayey loam	Wheat	Conventional
**c**	11	Loamy to highly loamy sand	Wheat	Conventional
**d**	9	Loamy sand	Rye	Organic
**e**	1	Slightly loamy to loamy sand	Rye	Conventional
**f**	20	Loamy sand	Potatoes	Conventional + irrigation

**Table 2:** Fertilization measures per area (personal correspondence).

Field	Date	Fertilizer Type	Amount/ha	N_verf._/ha
**a**	-	-	-	-
**b**	13.02.2023	Urea (46% N)	100 kg	46 kg
	16.03.2023	NPK (15/15/15)	100 kg	15 kg
	18.04.2023	Urea (46% N)	100 kg	46 kg
	13.05.2023	Urea (46% N)	100 kg	46 kg
**c**	14.02.2023	SSA 2023	180 kg	38 kg
	28.03.2023	KAS + MgO	300 kg	81 kg
	04.04.2023	Menure (Spring 2023)	25.27 m^3^	43 kg
**d**	-	-	-	
**e**	19.03.2023	NPK (15/15/15)	120 kg	15 kg
	22.04.2024	Urea (46% N)	120 kg	46 kg
**f**	21.04.2023	PPL 23	2.2 t	40 kg
	24.04.2023	Urea (46% N)	200 kg	92 kg

**Table 3:** Overview of the total of 1187 FarmLab measurements broken down by study area and month.

Field	Nov 22	Dec 22	Jan 23	Feb 23	Mar 23	Apr 23	May 23	Jun 23	Jul 23	Aug 23	Total
**a**	-	-	-	-	-	-	28	17	14	15	74
**b**	45	-	-	43	41	42	35	-	-	33	239
**c**	22	-	23	-	24	22	16	-	-	17	124
**d**	-	-	-	11	32	31	24	-	-	7	105
**e**	-	-	-	-	-	88	2	-	-	18	108
**f**	35	62	66	86	97	81	-	-	25	85	537
Total	102	62	89	140	184	264	105	17	39	175	1.187

**Table 4:** Sampling approaches applied to the individual study areas.

Area	Sampling Approach
**a**	Sampling of a specific measuring point (very small study area) *Time series analysis*
**b**	Sampling of predefined tiles in the study area *Correlation + cluster analysis*
**c**	
**d**	
**e**	
**f**	Randomized sampling of the entire study area *Geostatistical analysis*

**Table 5:** Descriptive statistics for location (a) with focus on measuring Nmin, P, K, Mg, SOC, moisture content, pH value, and soil temperature (soil temp.).

	Nmin [kg/ha]	P [mg/100 g]	K [mg/100 g]	Mg [mg/100 g]	SOC [%]	Moisture Content [%]	pH	Soil Temp. [°C]
*Count*	74	74	74	74	74	74	74	74
*Mean*	81.58	18.03	10.90	8.48	1.83	12.44	6.65	19.74
*Std*	23.44	5.26	1.02	1.89	0.46	3.74	0.25	3.45
*CV*	0.29	0.29	0.09	0.22	0.25	0.30	0.04	0.17
*Min*	44.00	7.50	9.00	3.50	1.00	7.60	6.10	10.70
*25%*	67.25	13.98	10.13	7.23	1.50	9.70	6.50	18.00
*50%*	77.00	17.40	10.90	8.45	1.70	11.20	6.65	20.10
*75%*	92.75	22.00	11.60	9.78	2.10	14.38	6.88	22.28
*Max*	165.00	34.30	13.90	13.00	3.30	21.20	7.10	25.50

**Table 6:** Descriptive statistics for field (f) in terms of time between two consecutive measurements [mm:ss] after clean-up break times (>15 min).

Count	Mean	Std	Min	25%	50%	75%	Max
*510*	04:04	01:34	01:44	03:09	03:37	04:21	14:33

## Data Availability

The original contributions presented in this study are included in the article. Further inquiries can be directed to the corresponding author.

## Supplementary Materials

The following supporting information can be downloaded at: https://www.mdpi.com/article/10.3390/s25247430/s1. S1: Online Resource 1: Parameters provided by the FarmLab cloud for each measurement (Stenon GmbH, 2024). S2: Online Resource 2: Results of Shapiro-Wilk tests on parameters Nmin, phosphorus, potassium and magnesium of location (a).

## Abbreviations

The following abbreviations are used in this manuscript:

AUF	Faculty for Agriculture, Civil and Environmental Engineering, Rostock University
SOC	Soil Organic Content
