# Supplementary material for: Spatial and Temporal Field-Scale Accuracy Assessment of a Multi-Sensor Spade for In Situ Soil Diagnostics: Performance and Limitations of the Stenon FarmLab for Precision Agriculture
Source: Sensors (Basel). 2025 Dec 6;25(24):7430. doi: 10.3390/s25247430 (PMC12737063; doi:10.3390/s25247430)
Supplement: Supplementary file 1 [file sensors-25-07430-s001.zip › sensors-3883248-supplementary.pdf]

S1: Online Resource1: Parameters provided by the FarmLab cloud for each measurement (Stenon GmbH, 2024)

Soil parameters	Unit	Measuring range		
Mineral nitrogen (N_{min})	kg / ha	0	–	252
Nitrate as nitrogen (NO_3-N)	kg / ha	0	–	252
Total nitrogen (N_{total})	%	0	–	0.5
Phosphorus (P)*	mg / 100 g	1.4	–	25
Potassium (K)*	mg / 100 g	3	–	25
Soil Moisture	%	0	–	30
pH value*		4.5	–	7.8
Soil Organic Carbon (SOC)*	%	0.3	–	3
Soil Organic Matter (SOM)*	%	0.5	–	5.2
**Carbon/Nitrogen (C/N) Ratio***		5	–	40
Magnesium (Mg)*	mg / 100 g	1.5	–	25
Soil Temperature*	°C	-10	–	50
Light (infrared)**	lx	0	–	10,000
Light (visible)**	lx	0	–	10,000
Air Temperature**	°C	-10	–	50
Air Humidity**	%	10 %	–	90 %
Air Pressure**	hPa	900	–	1,100
Volatile organic compounds**	Ohm	0	–	150,000,000
Carbon dioxide (CO_2)*	t			

* Parameters marked as "beta" are not certified by the DLG
** Parameters are physical measurements captured by the sensors

S2: Online Resource2: Results of Shapiro-Wilk tests on parameters Nmin, phosphorus, potassium and magnesium of location (a)

	Nmin [kg/ha]	*P [mg/100g]*	*K (beta) [mg/100g]*	*Mg [mg/100g]*
Statistic	0.917	0.977	0.975	0.993
p-value	0.00013	0.1988	0.1535	0.9789
